# Humidity‐Controlled Smart Window with Synchronous Solar and Thermal Radiation Regulation

**DOI:** 10.1002/advs.202506980

**Published:** 2025-06-04

**Authors:** Guozheng Li, Xiaofeng Jiang, Hafiz Asfahan, Siyuan Jia, Sujin Shao, Xiuqiang Li

**Affiliations:** ^1^ Key Laboratory for Intelligent Nano Materials and Devices of Ministry of Education and Institute for Frontier Science Nanjing University of Aeronautics and Astronautics Nanjing 210016 China

**Keywords:** smart window, solar heating, radiative cooling

## Abstract

Dynamic and continuous regulation of both solar and thermal radiation through windows is critical for enhancing building energy efficiency. However, achieving such adaptive regulations within a single window remains a formidable challenge. In this study, a humidity‐responsive polytetrafluoroethylene (PTFE)/ polyvinyl alcohol (PVA) composite membrane is developed with a nanoporous structure and integrated between two layers of glass to engineer a humidity‐controlled smart window. By modulating the humidity level between the glass layers, the smart window enables the dynamic and coordinated modulation of solar and thermal radiation via the reversible adsorption and desorption of water vapor by the PTFE/PVA composite. The results indicate that the humidity‐controlled smart window demonstrates superior capabilities in solar modulation (54.4%) and thermal radiation modulation (51.4%). Statistical analysis reveals that compared to ordinary windows and low emissivity (Low‐E) windows, PTFE/PVA windows reduce total annual energy consumption by ≈25% and 40%, respectively. This broadband dynamic continuous regulation mechanism may offer groundbreaking insights into the advancement of smart window technologies.

## Graphical abstract

A humidity‐responsive PTFE/PVA composite membrane with a nanoporous structure is developed and integrated between double glazing for the construction of smart windows. By modulating the interlayer humidity levels, this system achieves dynamic solar modulation (54.4%) and thermal radiation modulation (51.4%) via reversible water vapor adsorption/desorption within the PTFE/PVA composite.

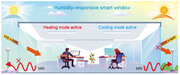

## 1 Introduction

The building sector significantly contributes to global energy consumption, accounting for 30% to 40% of the total.^[^ ^1^ ^]^ Approximately 50% of a building's total energy consumption is attributed to the energy demand of heating, ventilation, and air conditioning (HVAC) systems.^[^ ^2^ ^]^ Windows, which typically constitute 15% to 40% of the building envelope, are recognized as one of the most critical components affecting building energy efficiency.^[^ ^3^ ^]^ In the natural world, solar radiation (≈5800 K) transfers energy to the Earth as shortwave radiation, achieving heating, while the Earth dissipates heat to deep space (≈3 K) through long‐wavelength infrared (LWIR, *λ* ≈ 8−13 µm), achieving cooling.^[^ ^3^, ^4^ ^]^ This natural energy exchange mechanism forms the theoretical foundation for developing new smart window technologies. A window with high solar transmittance and low mid‐infrared emissivity can achieve effective solar heating. Conversely, a window with high solar reflectance and high mid‐infrared emissivity can facilitate cooling via radiative cooling.^[^ ^5^ ^]^ Focusing solely on a single mechanism, such as solar heating or radiative cooling, can lead to imbalanced thermal management due to rapid changes in the external environment, resulting in overheating or overcooling inside buildings, which not only fails to achieve energy savings but may also increase energy consumption.^[^ ^6^ ^]^ Therefore, developing smart windows with dynamic adaptability and multimodal regulation capabilities is essential for achieving energy‐efficient buildings.^[^ ^7^ ^]^

Existing smart windows are primarily driven by thermal,^[^ ^8^ ^]^ electronic,^[^ ^9^ ^]^ or mechanical^[^ ^10^ ^]^ means. Most of these approaches limit the regulatory spectra to solar or mid‐infrared. In recent years, while a few studies have demonstrated broadband regulation of solar spectra and mid‐infrared spectra using thermal stimuli,^[^ ^11^ ^]^ electric drive,^[^ ^6a^ ^]^ or organic solvents,^[^ ^12^ ^]^ these techniques either lack continuous regulation capability or exhibit insufficient regulation. This limitation constrains the adaptability and efficiency of smart windows in responding to varying climate conditions during practical applications.

**Figure 1:**
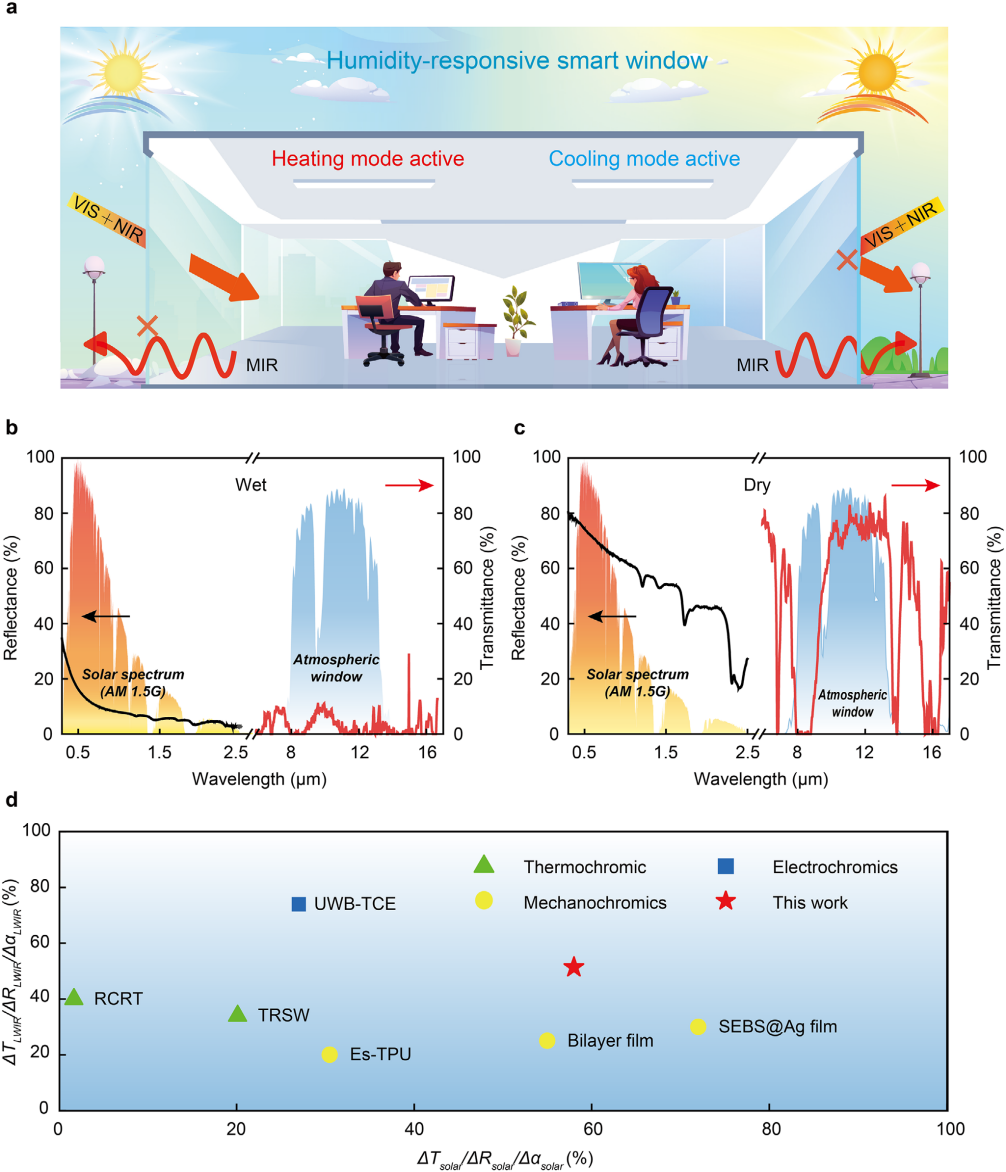
Performance of PTFE/PVA composite membrane. a) Optical schematics of smart windows equipped with PTFE/PVA composite membrane in the dry and wet modes. The left and right smart windows represent the wet and dry modes, respectively. b) Solar reflectance and thermal infrared transmittance of the composite membrane in the dry mode. c) Solar reflectance and thermal infrared transmittance of the composite membrane in the wet mode. d) Broadband dynamic photothermal regulation capabilities of smart materials reported in the literature. (Thermochromic,^[^ ^13^ ^]^ Electrochromics^[^ ^14^ ^]^ or Mechanochromics^[^ ^15^ ^]^).

To address the limitations of current technologies, we developed a humidity‐responsive polytetrafluoroethylene (PTFE)/ polyvinyl alcohol (PVA) composite membrane with a nanoporous structure and integrated it between two layers of glass to engineer a humidity‐controlled smart window. By modulating the humidity level between the glass layers, the smart window enables the dynamic and coordinated modulation of solar and thermal radiation via the reversible adsorption and desorption of water vapor by the PTFE/PVA composite. As illustrated in **Figure** **1**a–c, in the dry mode, it achieves a high solar reflectance (*R_sola_*_r_ = 66.4%), effectively preventing solar heating, and a high long‐wave infrared transmittance (*T_LWIR_* = 54.6%), enabling efficient indoor heat dissipation. Conversely, in the wet mode, the membrane exhibits a low solar reflectance (*R_solar_* = 12.0%), maximizing solar energy utilization for heating, and a low long‐wave infrared transmittance (*T_LWIR_* = 3.2%), significantly reducing heat loss. The long‐wavelength infrared emissivity of the composite film was measured to be 44.87% in the dry state and 83.72% in the wet state, respectively (Figure S1, Supporting Information). Statistical analysis showed that, compared to ordinary windows and low emissivity (Low‐E) windows, PTFE/PVA windows reduced total annual energy consumption by ≈25% and 40%, respectively. Compared to existing broadband smart windows reported in the literature (e.g., Thermochromic,^[^ ^13^ ^]^ Electrochromics,^[^ ^14^ ^]^ or Mechanochromics^[^ ^15^ ^]^) (Figure 1d), our designed smart window demonstrates superior performance, with an exceptional solar modulation capability (*ΔR_solar_* = 54.4%) and thermal modulation capability (*ΔT_LWIR_* = 51.4%). Moreover, it possesses the advantages of a simplistic structure and being pollution‐free, thereby demonstrating superior practicality.

## 2 Results and Discussion

### 2.1 Photothermal Switching Mechanism of PTFE/PVA Membrane

**Figure 2:**
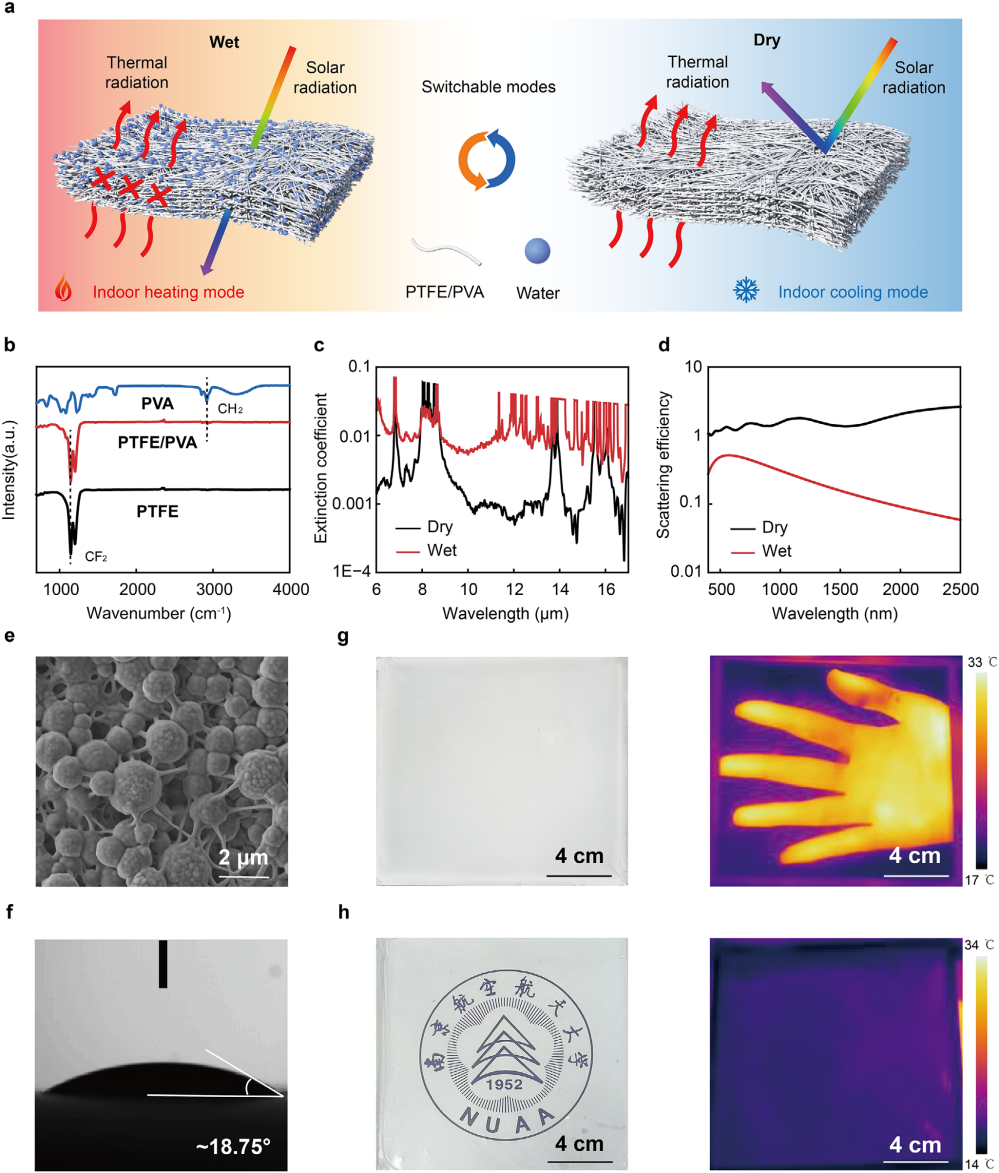
Photothermal switching mechanism and membrane properties of PTFE/PVA composite membrane. a) Optical switching behavior of PTFE/PVA membranes in the solar and thermal radiation spectra in dry and wet modes. The dry and wet modes are used for cooling and heating, respectively. b) Comparison of characteristic peaks of PTFE/PVA composite membrane with PTFE and PVA. c) Effective electromagnetic extinction coefficients of the PTFE/PVA composite membrane in dry and wet conditions, respectively. d) Scattering efficiencies of the PTFE/PVA composite membrane in dry and wet conditions, respectively. e) SEM image of the PTFE/PVA composite membrane. f) Water contact angle of the PTFE/PVA composite membrane. (g and h) Optical and infrared images of the PTFE/PVA composite membrane in dry g) and wet h) modes, respectively.

In the solar spectrum range (*λ* ≈ 0.3–2.5 µm), the pores within the membrane are filled with air in the dry state (**Figure** **2**a, Left). Due to the significant refractive index contrast between the membrane material and air (Figure 2d), sunlight undergoes substantial scattering at the pore boundaries, resulting in a white appearance of the membrane.^[^ ^12^, ^16^ ^]^ When the air within the pores is replaced by water (Figure 2a, Right), the refractive index of the membrane becomes more similar to that of water, significantly reducing scattering efficiency and thereby enhancing transmittance. In the thermal infrared spectrum (*λ* ≈ 8–13 µm), polytetrafluoroethylene (PTFE) dominates the membrane's optical properties. The CF₂ bonds within PTFE exhibit vibrational excitation near 8.3 µm (Figure 2b), leading to a low electromagnetic extinction coefficient *K* (Figure 2c).^[^ ^16^, ^17^ ^]^ Consequently, in the dry state, the membrane demonstrates high transmittance in the thermal infrared range. However, when the pores are filled with water, the high infrared absorption characteristic of water results in a markedly increased electromagnetic extinction coefficient *K* (Figure 2c), causing the membrane to exhibit extremely low infrared transmittance.

### 2.2 Preparation and Properties of PTFE/PVA Membrane

The preparation process of PTFE/PVA membranes involves the precise mixing of PTFE with an aqueous PVA solution at a predetermined ratio, followed by electrospinning to form the membrane and subsequent low‐temperature sintering at 120 °C. Research has demonstrated that both the PVA concentration in the spinning solution and the thickness of the membrane significantly influence spectral radiation performance (Figure S2, Supporting Information). Initially, composite membranes with a thickness of ≈15 µm were prepared by controlling the electrospinning time to 5 h. Subsequently, the effect of varying the PTFE/PVA spinning solution ratios on material properties was investigated. It was found that when the mass ratio of PTFE solution (60 wt.%) to PVA solution (15 wt.%) was set at 6:1, 8:1, 10:1, and 11:1, the corresponding solar reflectance modulation amplitudes were 63.36%, 51.35%, 41.76%, and 16.89%, respectively, while the long‐wavelength infrared transmittance modulation amplitudes were 25.93%, 38.91%, 58.16%, and 37.77%, respectively. Furthermore, when the mass ratio of the spinning solution reached 12:1, the electrospinning process failed due to insufficient PVA content, resulting in an inability to produce any membrane. Therefore, considering the modulation capabilities in both the solar and long‐wavelength infrared bands, the spinning solution with a 10:1 ratio was selected. Subsequently, the thickness was optimized by controlling the electrospinning time to fabricate composite membranes with controlled thicknesses of 16, 26, 37, and 43 µm. These samples exhibited distinct optical properties, with solar reflectance modulation amplitude values of 40.22%, 54.35%, 55.73%, and 58.68%, respectively, and long‐wave infrared transmittance values of 53.47%, 51.40%, 33.20%, and 30.41%, respectively. Testing revealed that at a thickness of 26 µm, the membrane achieved optimal modulation performance in both the solar and long‐wavelength infrared bands, with values of 54.4% and 51.4%, respectively.

The electrospinning process integrates PTFE particles within a fibrous PVA matrix, forming a porous network (Figure 2e). This microstructure confers exceptional hydrophilicity to the membrane, as evidenced by a water contact angle of only 18.75° (Figure 2f). The interstitial pores between fibers facilitate rapid water infiltration, making water an ideal medium for regulating the transition between dry and wet states of the membrane. Dynamic water vapor sorption analysis revealed that the fabricated PTFE/PVA layer exhibits excellent hygroscopic properties. As humidity increases, the water content within the material gradually rises, eventually reaching 11.48 mg g^−1^ in the sample. Conversely, a decrease in humidity leads to a corresponding reduction in water content, with the sample returning to 0.13 mg g^−1^ (Figure S3, Supporting Information).

The fabricated samples, measuring 20 × 20 cm^2^, exhibit potential for large‐scale production. The optical transparency of the PTFE/PVA membrane exhibits marked differences between its dry and wet states (Figure 2g,h). In the wet state, the high transmittance enables a clear visualization of the colored Nanjing University of Aeronautics and Astronautics (NUAA) logo, whereas in the dry state, the logo is completely obscured. Furthermore, infrared imaging demonstrates that in the wet state, the membrane effectively blocks thermal radiation from a hand, while in the dry state, thermal radiation readily penetrates the membrane. After parameter optimization, the PTFE/PVA membrane demonstrated superior performance in both the solar spectrum (*ΔR_solar_* = 54.4%) and the thermal infrared spectrum (*ΔT_LWIR_* = 51.4%).

### 2.3 Continuous Regulation Performance of Humidity Response Smart Window

**Figure 3:**
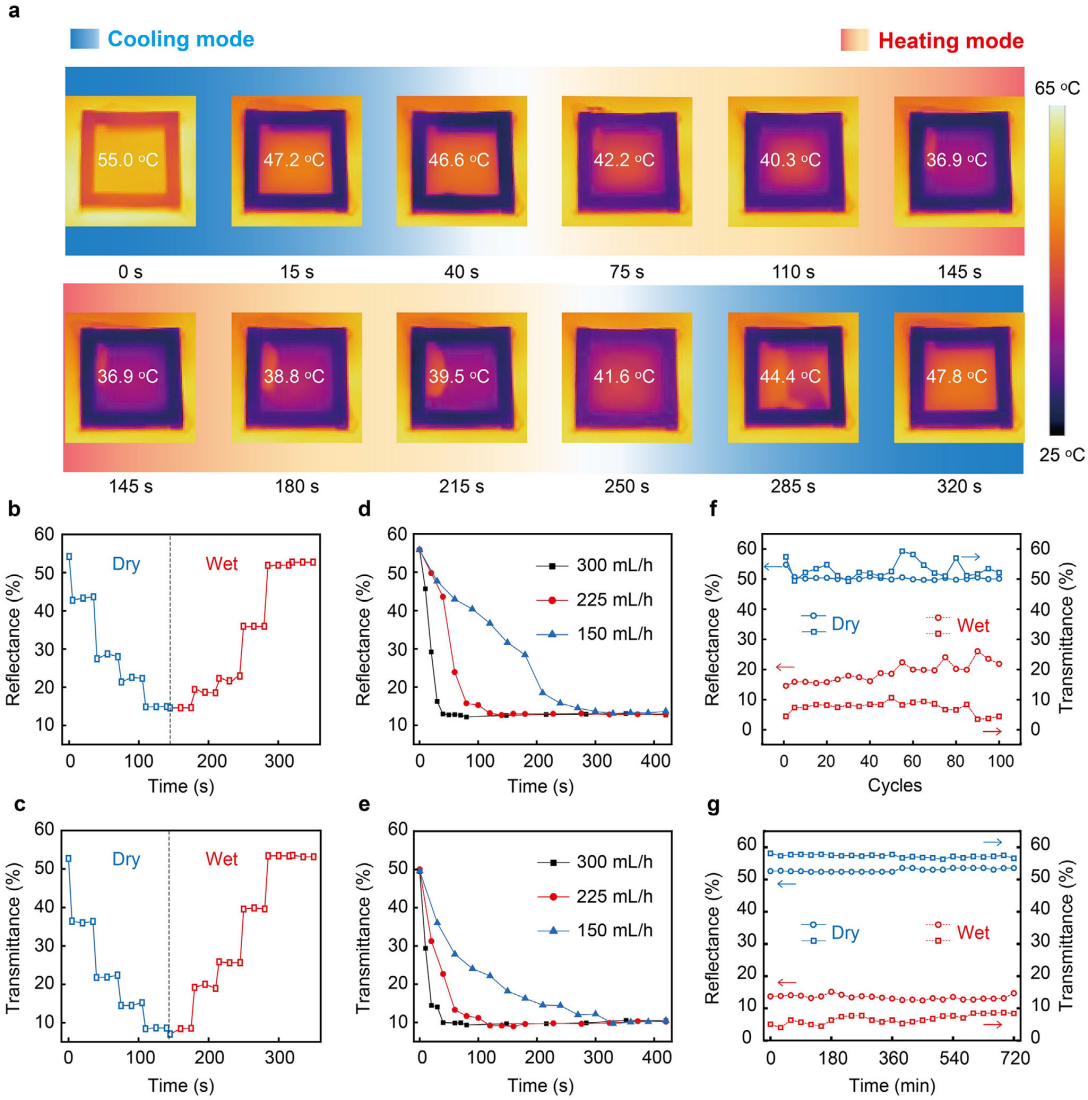
Continuous regulation performance of the PTFE/PVA‐based moisture‐response smart window. a) The upper infrared images (0–145 s, 55.5 °C, 47.2 °C, 46.6 °C, 42.2 °C, 40.3 °C, 36.9 °C) and lower infrared images (145–320 s, 36.9 °C, 38.8 °C, 39.5 °C, 41.6 °C, 44.4 °C, 47.8 °C) of the window from dry mode to wet mode and from wet mode to dry mode, respectively. b) Continuous variation of the reflectance of the window in the solar band from dry to wet and then to dry modes. c) Continuous variation of the mid‐infrared transmittance of the window from dry to wet and then to dry modes. d and e) Effect of humidification rate on the solar band reflectance (d) and mid‐infrared transmittance (e) of the window, respectively. f) Cycling stability of the window between dry and wet modes. g) Long‐term stability of the window in dry and wet modes.

To validate the dynamic and continuous tunability of the humidity‐controlled smart window, a PTFE/PVA membrane was integrated into a testing device (detailed structure shown in Figure S4, Supporting Information). The PTFE/PVA smart window was subjected to controlled environmental testing in a climate chamber maintained at 25 °C with relative humidity levels of 40%, 60%, 80%, and 95 ± 3%. Solar reflectance and long‐wave infrared transmittance were measured at 10‐min intervals over a 30‐min period (Figure S5, Supporting Information). After 30 min of operation, the smart window exhibited merely a 5% decrease in both solar transmittance and long‐wave infrared transmittance. By introducing humid or dry air through a pipeline, dynamic transitions between the wet and dry states of the membrane can be effectively achieved. Specifically, humid air was introduced at a flow rate of 300 mL min^−1^ to gradually transition the membrane from its dry state to its wet state. Subsequently, dry air with 20% relative humidity was introduced at a flow rate of 2.5 L min^−1^ to revert the membrane to its original dry state. Each humidification and dehumidification process lasted for 5 s, followed by a 30‐s interval during which no external conditions were applied. As illustrated in **Figure** **3**a, infrared images captured the state transitions of the device at each modulation stage, demonstrating remarkable continuous tunability across different stages. Figure 3b,c quantify the performance metrics at each stage. This further confirms that the PTFE/PVA‐based humidity‐controlled smart window exhibits excellent continuous tunability. Additionally, we conducted a systematic study on the effects of gas flow rate and humidity on switching response speed (Figure 3d,e; Figure S6, Supporting Information). The results demonstrate that an increase in the humid air flow rate markedly reduces the time required for *R_solar_* and *T_LWIR_* to achieve their stable equilibrium states (Figure 3d,e). Specifically, when the humid air flow rate increased from 150 to 300 mL h^−1^, the time required to reach the final state decreased from 300 to 50 s. In contrast, higher flow rates ranging from 1.5 to 2.5 mL min^−1^, or lower humidity levels from 80% to 20% during dry air introduction, resulted in a more rapid recovery of *R_solar_* and *T_LWIR_* to their dry‐state values (Figure S6, Supporting Information).

Furthermore, the durability of the device was evaluated through cyclic testing. The spectral characteristics of the humidity‐controlled smart window were measured every five cycles (see Figure S7, Supporting Information). After 100 dry‐wet cycles (Figure 3f), the dry‐state solar reflectance (*R_solar, dry_*) decreased from 54.8% to 50.1%, while the wet‐state solar reflectance (*R_solar, wet_*) increased from 14.6% to 21.9%. For thermal long‐wave infrared radiation (*T_LWIR_*), the dry‐state long‐wavelength infrared transmittance (*T_LWIR, dry_*) decreased from 57.4% to 52.1%, whereas the wet‐state long‐wavelength infrared transmittance (*T_LWIR, wet_*) remained stable at 4.4%. To further assess the long‐term stability of the device, extended tests were conducted under both dry and wet conditions (Figure 3g). The spectral characteristics of the window were recorded every 30 min (see Figure S8, Supporting Information). The results indicate that the device‐maintained stability for at least 12 h in both states following the sealing process, with performance degradation of merely 1%. These findings demonstrate that the PTFE/PVA‐based optical switching device exhibits excellent performance consistency and long‐term stability, making it highly suitable for practical applications.

### 2.4 Indoor Temperature Regulation Tests of Humidity Response Smart Window

**Figure 4:**
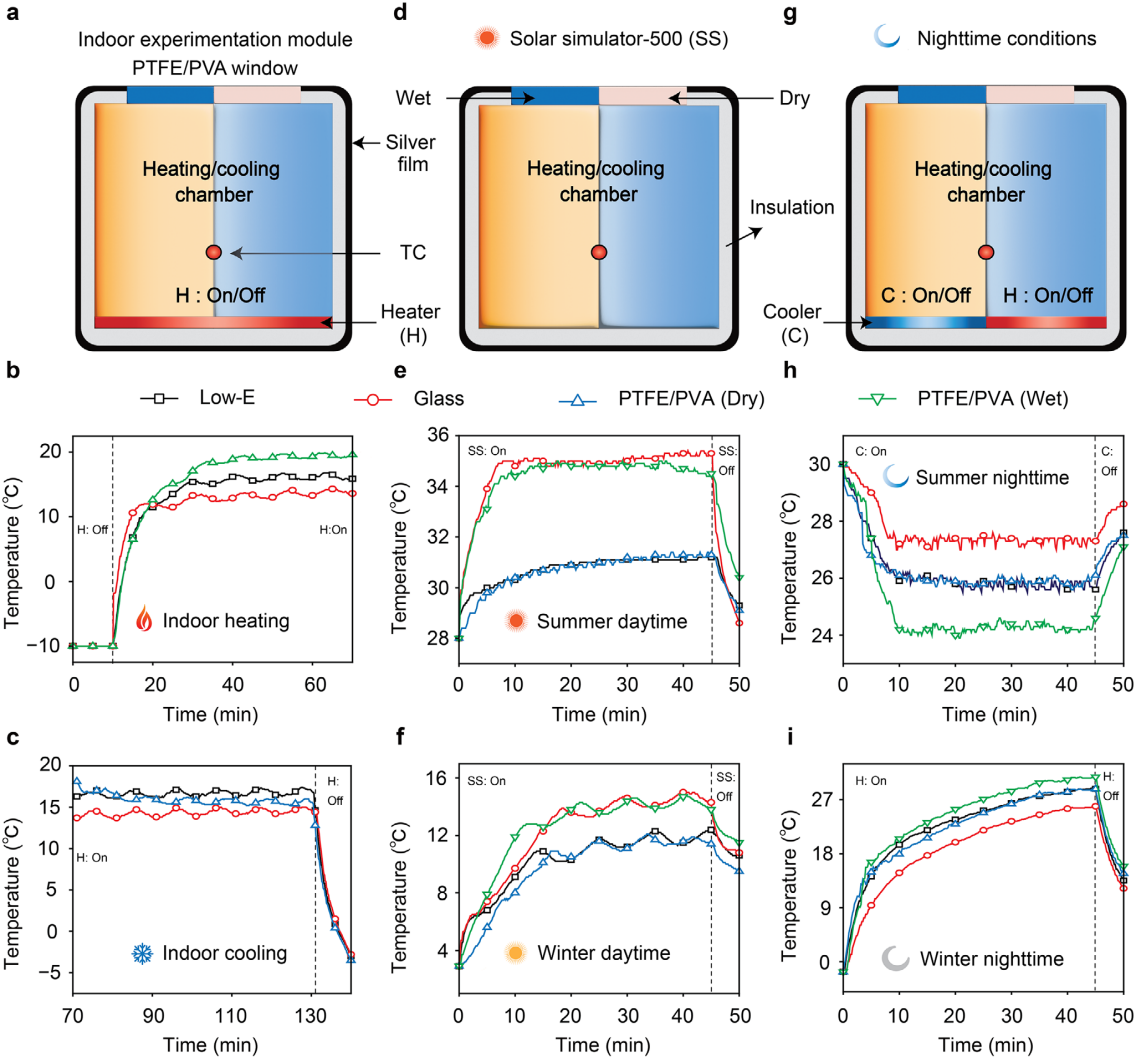
Thermal management testing of chambers with different windows (low‐E window, glass window and PTFE/PVA window with dry and wet modes). a) Schematic diagram of the setup for thermal management testing. b and c) Indoor air temperature records of the chambers with different windows under wet mode (b) and dry mode (c). d) Schematic diagram of the daytime testing setup. e) Indoor air temperature records in simulated summer daytime. f) Indoor air temperature records in simulated winter daytime. g) Schematic diagram of the nighttime testing setup. h) Indoor air temperature records in simulated summer nighttime. i) Indoor air temperature records for simulated winter nighttime. TC represents the thermocouple in the setup. “Indoor heating” corresponds to the wet mode of the PTFE/PVA window. “Indoor cooling” corresponds to the dry mode of the PTFE/PVA window.

To evaluate the indoor temperature regulation capabilities, we designed a sealed chamber with dimensions of 10 cm × 10 cm × 10 cm, featuring a window measuring 3.5 cm × 3.5 cm. In a comparative study, three types of windows were utilized: ordinary glass, commercial Low‐E glass, and PTFE/PVA window. The optical properties of these three window types are summarized in Table S1 (Supporting Information). The thermoresponsive behaviors of these windows were assessed by monitoring the temporal variations in indoor air temperature. During the experiments, each test chamber was initially set to −10 °C and subsequently heated using an internal heater with a constant output power of 3.2 W (**Figure** **4**a). The resultant changes in indoor air temperature were recorded. In the initial wet state mode (Figure 4b), the PTFE/PVA window enabled the indoor air temperature to reach ≈19.8 °C, outperforming both Low‐E window (17 °C) and ordinary glass window (14.6 °C). When switched to the dry state mode (Figure 4c), the indoor air temperature decreased from 19.8 °C to around 15.6 °C, which was approximately 1.4 °C lower than that of Low‐E window (17 °C). This phenomenon primarily arises from the distinct thermophysical properties of the PTFE/PVA window in different states. In the wet state, the window exhibits both low long‐wave infrared transmittance (≈4.4%) and significantly reduced thermal conductivity (≈0.0295 W/(m·K)) compared to Low‐E window (≈0.747 W/(m·K)), resulting in superior thermal insulation performance and the highest indoor air temperature. Conversely, in the dry state, the long‐wave infrared transmittance of the PTFE/PVA window increases substantially to ≈57.4%. When compared with the high thermal reflectivity of Low‐E window (≈66.8%), this property allows more efficient heat dissipation to the external environment (‐10 °C), thereby leading to a lower indoor air temperature than that achieved with Low‐E window. These results indicate that the PTFE/PVA window exhibits superior thermal insulation performance and notable temperature regulation capabilities, with a difference of ≈4.2 °C during the transition from wet to dry mode.

A solar simulator and an environmental temperature control system were utilized to replicate typical summer and winter daytime climatic conditions (Figure 4d). In the simulated summer daytime scenario, the ambient temperature was set at 28 °C, and the irradiance of the solar simulator was adjusted to 1000 W m^−^^2^. As shown in Figure 4e, the indoor temperature regulated by the PTFE/PVA window gradually increased to 31.3 °C in dry mode and to 34.5 °C in wet mode, respectively. By contrast, the indoor temperature under the Low‐E window reached 31.2 °C, while that of the ordinary glass window rose to 35.3 °C. These findings suggest that during summer daytime, both the PTFE/PVA window in dry mode and the Low‐E window achieve relatively lower indoor temperatures compared to the ordinary glass window. This phenomenon is closely associated with the optical properties of the windows. Specifically, in dry mode, the solar reflectance of the PTFE/PVA window is comparable to that of the Low‐E window (both ≈54.8%), resulting in similar indoor temperature performance. In the simulated winter daytime scenario, the ambient temperature was set to 2.9 °C, and the irradiance of the solar simulator was adjusted to 800 W m^−^^2^. As illustrated in Figure 4f, the indoor temperature regulated by the PTFE/PVA window gradually increased to 11.4 °C in dry mode and to 13.8 °C in wet mode. In contrast, the indoor temperature rose to 12.4 °C under the Low‐E window and to 14.3 °C under the ordinary glass window. We also conducted a simulation experiment on indoor temperatures in winter by setting the solar irradiance at 400 W m^−^^2^ based on sunlight intensity data for January in Nanjing (Figure S9, Supporting Informationa, obtained from European Centre for Medium‐Range Weather Forecasts). The test results showed that the temperature trend was similar to that at 800 W m^−^^2^ (Figure S9b, Supporting Information). The highest temperature recorded was 9.9 °C for the glass window, 9.7 °C in wet mode, 7.5 °C for the Low‐E window, and 7.4 °C in dry mode. These results indicate that during winter daytime, both the ordinary glass window and the PTFE/PVA window in wet mode achieve higher indoor temperatures. This phenomenon is closely associated with the optical properties of the windows. Specifically, in wet mode, the PTFE/PVA window has a solar transmittance of 77.7%, slightly lower than the 87.5% of the ordinary glass window. However, the lower thermal conductivity of the PTFE/PVA window compensates for the reduced solar gain caused by its lower solar transmittance, resulting in comparable indoor temperatures for both types of windows. Consequently, the PTFE/PVA window in wet mode and the ordinary glass window exhibit the highest indoor temperatures.

By integrating a 3 W polyimide film heating element or a 13 W Peltier cooling element at the bottom of the chamber, experiments were conducted to simulate summer nighttime cooling and winter nighttime heating scenarios (Figure 4g). In the summer nighttime experiment (ambient temperature 30 °C), upon activation of the cooler, the indoor temperatures under the PTFE/PVA window in dry and wet modes gradually decreased to 25.6 and 24.6 °C, respectively. In contrast, the indoor temperature regulated by the Low‐E window decreased to 25.6 °C, while that of the ordinary glass window decreased to 27.3 °C. This phenomenon is primarily attributed to the varying thermal conductivity and thermal radiation performance of the different window types.^[^ ^18^ ^]^ Specifically, in wet mode, the PTFE/PVA window's low thermal transmittance (≈4.4%) and low thermal conductivity (≈0.0295 W/(m·K)) play crucial roles in minimizing the heating effect from the outdoor environment, thereby maintaining a lower indoor temperature. Although the thermal transmittance of the PTFE/PVA window in dry mode (57.4%) is higher than that of the Low‐E window (0.6%), its lower thermal conductivity (≈0.0273 W/(m·K)) compensates for the increased heat loss due to higher thermal transmittance, resulting in comparable indoor temperatures between the two. The ordinary glass window, characterized by its higher thermal emissivity (≈89.8%) and thermal conductivity (≈0.733 W/(m·K)), results in the highest indoor temperature (Figure 4h). In the winter nighttime experiment (ambient temperature −1.7 °C), after activating the heater, the indoor temperatures under the PTFE/PVA window increased to 28.8 °C in dry mode and 30.8 °C in wet mode, respectively. In contrast, the indoor temperature regulated by the Low‐E window rose to 28.8 °C, while that of the ordinary glass window reached only 25.9 °C. This phenomenon can be attributed primarily to the distinct thermal conductivity and thermal radiation properties of the different window types. Specifically, the PTFE/PVA window's low thermal transmittance (≈4.4%) and low thermal conductivity (≈0.0295 W/(m·K)) are key factors contributing to its superior heating effect. Although the thermal transmittance of the PTFE/PVA window in dry mode (57.4%) is higher than that of the Low‐E window (0.6%), its lower thermal conductivity (≈0.0273 W/(m·K)) compensates for the heat loss due to higher thermal transmittance, resulting in comparable indoor temperatures between the two. The ordinary glass window, with its higher thermal emissivity and thermal conductivity, led to the lowest indoor temperature (Figure 4i). These experimental results demonstrate that the PTFE/PVA‐based window can achieve adaptive and reversible switching of solar and thermal transmittance, exhibiting outstanding indoor temperature regulation capabilities under varying weather conditions, including summer and winter, as well as daytime and nighttime scenarios. Moreover, the temperature regulation performance of typical electrochromic smart windows, thermochromic smart windows, and humidity‐controlled smart windows was systematically summarized, as detailed in Table S2 (Supporting Information).

We also conducted outdoor temperature measurements. The experiment was carried out in Nanjing, China on May 11, 2025 (Figure S10a, Supporting Information). Figure S10b (Supporting Information) illustrates the measured solar irradiance, while Figure S10c (Supporting Information) depicts the recorded ambient temperature and relative humidity. Figure S10d (Supporting Information) provides the temperature profiles of ordinary glass, Low‐E window, PTFE/PVA window in dry mode, and PTFE/PVA window in wet mode, with their respective peak temperatures reaching 51, 41.95, 38.17, and 50.58 °C. This thermal behavior can primarily be attributed to the following factors: The ordinary glass window exhibited the highest solar transmittance, followed by the wet mode configuration, leading to the maximum temperature peak for the glass window. Both the Low‐E window and the dry mode demonstrated similar solar reflectivity; however, the inward‐facing low‐emissivity coating of the Low‐E window effectively suppressed heat exchange between the chamber and the environment. The dry mode configuration showed higher infrared transmittance, enhancing environmental heat dissipation and consequently resulting in the lowest temperature peak among all test conditions.

### 2.5 Energy‐Saving Simulation of Humidity Response Smart Window

**Figure 5:**
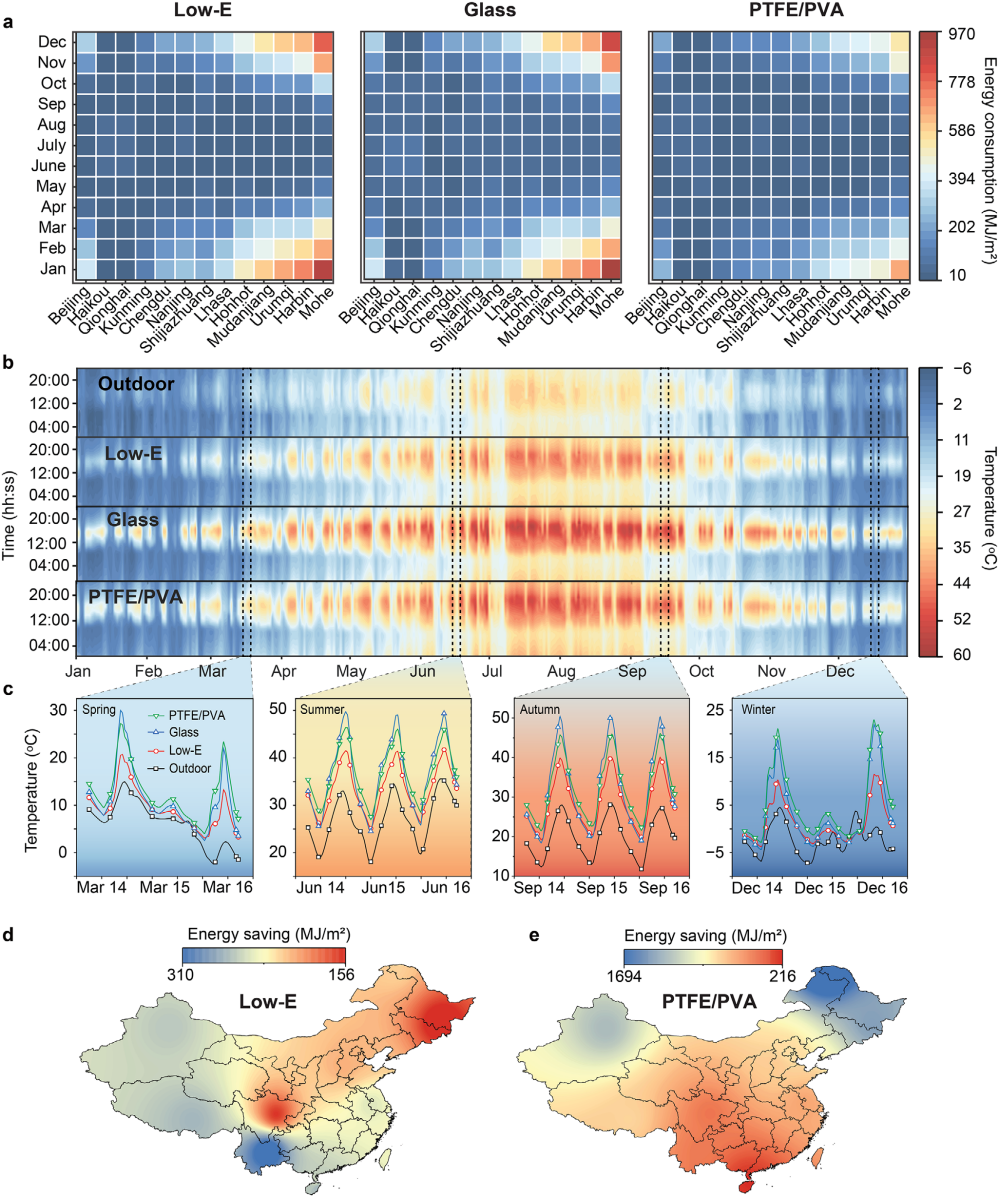
Performances and energy‐saving simulations of different windows (Low‐E window, glass window and PTFE/PVA window). a) Monthly energy consumption for Low‐E window, glass window, and PTFE/PVA window in 13 cities. b) Indoor annual temperature variation in Beijing for different windows. c) Indoor temperature variation over three consecutive days in Beijing during March, June, September and December. d) Year‐round energy savings map in China using Low‐E window. e) Year‐round energy savings map in China using PTFE/PVA window.

To evaluate the indoor temperature regulation performance of PTFE/PVA windows, a comprehensive simulation was conducted using the EnergyPlus platform. The analysis integrated climate data from Beijing's four distinct seasons. Beijing was chosen due to its pronounced annual temperature fluctuations, with summer temperatures frequently surpassing 30 °C and winter temperatures often dropping below −8 °C. The results were compared against those of ordinary glass windows and Low‐E windows. Assuming no additional internal heat sources, the performance for a single‐story house (refer to Figure S11, Supporting Information) was calculated based on the optical properties of the three different window types (see Table S1, Supporting Information). As shown in **Figure** **5**a, the simulation conducted in 13 cities demonstrated that, compared to ordinary glass windows, PTFE/PVA windows exhibited lower energy consumption throughout the year. When compared to Low‐E windows, PTFE/PVA windows showed lower energy consumption during the colder months (January, February, March, April, October, November, and December), while exhibiting comparable energy consumption during the warmer months (May, June, July, August, and September). As illustrated in Figure 5b, the simulation results for the annual temperature variation in Beijing demonstrate that PTFE/PVA window exhibit effective temperature regulation throughout the year. The details are as follows, in December (winter) (Figure 5c), the PTFE/PVA window achieved the highest daytime indoor temperature due to its high solar transmittance and low thermal conductivity, which enables it to effectively capture and retain solar heat. Conversely, Low‐E windows, characterized by lower solar transmittance, resulted in the lowest indoor temperatures. During nighttime, both PTFE/PVA and Low‐E windows maintained comparable indoor temperatures owing to their low thermal transmittance and high thermal reflectance. In contrast, ordinary glass windows exhibited lower indoor temperatures due to their relatively higher thermal transmittance and lower thermal reflectance. In March (spring), June (summer) and September (autumn) (Figure 5c), Low‐E windows demonstrated the lowest daytime indoor temperatures primarily because of their reduced solar transmittance, followed by PTFE/PVA windows and then glass windows. At night, the temperature performance of all three types of windows mirrored the trends observed in December, reflecting consistent patterns in thermal transmittance and reflectance. These findings indicate that PTFE/PVA windows, with their broad‐spectrum modulation capabilities, offer superior temperature regulation across different seasonal conditions, providing enhanced thermal insulation during winter and a cooling effect during summer compared to ordinary glass windows.

Based on the Koppen‐Geiger climate classification system,^[^ ^19^ ^]^ this study evaluates the performance of standard windows, Low‐E windows, and PTFE/PVA windows in maintaining an indoor temperature of 26 °C with the introduction of HVAC systems. The results demonstrate that Low‐E windows exhibit significant energy‐saving advantages in low‐latitude hot regions (Figure 5d). This is due to the low solar transmittance of Low‐E windows, which effectively prevents solar heating while their low infrared emissivity effectively isolates the exchange of heat radiation between the interior and exterior. As illustrated in Figure 5e, the results demonstrate that PTFE/PVA windows exhibit significant energy‐saving advantages in high‐latitude cold regions. This is primarily attributed to their efficient capture of solar heat during winter and their superior insulation performance at night, which substantially reduces heating energy demands. Furthermore, a statistical analysis of 13 representative cities in China revealed that the annual total energy consumption of PTFE/PVA windows was approximately 25% lower than that of ordinary glass windows and 40% lower than that of Low‐E windows. With their unique photothermal control capabilities, PTFE/PVA windows provide an exceptional energy‐saving solution for buildings in various climate regions, particularly in cold areas and cities with significant year‐round climate variations, thereby showcasing remarkable energy‐saving potential.

## 3 Conclusion

We have engineered a humidity‐controlled smart window by developing a humidity‐responsive PTFE/PVA composite material with nanopore structure. Through precise humidity control, this PTFE/PVA composite window achieves continuous modulation across a broadband spectral range, with solar reflectance modulation reaching up to 54.4% and thermal transmittance modulation of 51.4%. These features enable superior indoor temperature regulation under varying seasonal conditions. Statistical analysis indicates that, compared to conventional windows and Low‐E windows, the PTFE/PVA smart window reduces total annual energy consumption by ≈25% and 40%, respectively. Given its efficient and continuous regulatory performance, outstanding stability, and cost‐effectiveness, this innovative smart window presents a promising solution for building energy conservation.

## Conflict of Interest

The authors declare no conflict of interest.

## Author Contributions

X.L. conceived the idea, designed the experiments, and supervised the research project. G.L. conducted the experiments. G.L. and H.A. performed the simulation. G.L., X.J., S.S., and S.J. analyzed the data. G.L., X.J., and S.J. wrote the paper. All authors contributed to the experiments and discussion of the manuscript.

## Supporting information



Supporting Information

## Data Availability

The data that support the findings of this study are available from the corresponding author upon reasonable request.
