# Supplementary material for: Humidity‐Controlled Smart Window with Synchronous Solar and Thermal Radiation Regulation
Source: Adv Sci (Weinh). 2025 Jun 4;12(33):e06980. doi: 10.1002/advs.202506980 (PMC12412564; doi:10.1002/advs.202506980)
Supplement: Supplementary file 1 — Supporting Information [file ADVS-12-e06980-s001.docx]

**Supplementary Information for**

**Humidity-controlled Smart Window with Synchronous Solar and Thermal Radiation Regulation**

Guozheng Li, Xiaofeng Jiang, Hafiz Asfahan, Siyuan Jia, Sujin Shao, Xiuqiang Li*

Key Laboratory for Intelligent Nano Materials and Devices of Ministry of Education, and Institute for Frontier Science, Nanjing University of Aeronautics and Astronautics, Nanjing 210016, China

*Corresponding author.

Email: [xiuqiang.li@nuaa.edu.cn](mailto:xiuqiang.li@nuaa.edu.cn)

**Methods**

**Fabrication of PTFE/PVA composite membrane and Devices.**

The PTFE/PVA composite membrane was directly fabricated onto polyethylene films via an electrospinning process. Subsequently, the membrane was immersed in anhydrous ethanol and dried at 120 °C to enhance its bonding strength. By varying the electrospinning duration, PTFE/PVA composite membranes with different thicknesses were prepared. Finally, the membrane was integrated into a device (Detailed structure is shown in Figure S3) for testing. The PTFE commercial aqueous dispersion (60 wt% solid content, viscosity 25 mPa·s) was procured from Dongguan Colord Innovative Technology Co., Ltd., while the polyvinyl alcohol (molecular weight 44,050 Da) was obtained from Shanghai Aladdin Biochemical Technology Co., Ltd.

**Material Characterizations**.

Spectral measurements in the solar range (0.3 to 2.5 μm) were performed using a UV-Vis-NIR spectrophotometer (UH4150) equipped with an integrating sphere. Spectra in the mid-infrared range (6 to 17 μm) were acquired using a Fourier transform infrared spectrometer (iS50 FT-IR) fitted with a gold-coated integrating sphere from PIKE Technologies. Both solar and long-wave infrared spectral measurements were performed on the integrated PE-PTFE/PVA composite membrane system. Surface groups of the composite membranes were characterized by FT-IR spectroscopy using a Nicolet RaptIR FT-IR Microscope. Effective electromagnetic extinction coefficients k was derived from transmittance measurements following the technique described in reference 36. Refractive indices of the materials were determined using an ellipsometer (J.A. Woollam IR-VASE, USA), while scattering efficiencies of the samples were simulated using Lumerical's FDTD Solutions 8.6.1 software. Morphological analysis of the samples was conducted via Scanning Electron Microscopy (Sigma300, SEM). Contact angles were measured using a contact angle meter (JC2000DS). Infrared images were captured by an infrared camera (Fotric 325) with the emissivity set to 0.98. The water vapor adsorption and desorption capacity of the material was evaluated using a dynamic water vapor sorption analyzer (3P garviSorb). Environmental humidity effects on smart window performance were investigated using a constant temperature and humidity incubator (HWS-30B) for precise humidity control. Thermal conductivity of various materials was assessed using a thermal conductivity meter (DRL-III). Integrated solar reflectance (*R_Solar_*) and thermal infrared transmittance (*T_LWIR_*) were calculated from spectral data through weighted integration based on the normalized ASTM G173 solar spectrum or the blackbody spectrum at 25 °C.

**Preparation of temperature management tests.**

The test chamber was constructed from 2 cm thick white extruded polystyrene (XPS) panels, with an internal volume of 10 cm × 10 cm × 10 cm. A 3.5 cm × 3.5 cm aperture on one side facilitated the installation of various window materials. The light source for simulating daytime conditions was provided by a solar simulator (Solar-500). An internal thermal load was generated by a PI film heating element (10 cm × 10 cm), which was affixed to the interior surface of the XPS base wall. Additionally, a Peltier cooler (10 cm × 10 cm) was attached to the same surface to simulate cooling conditions. A T-type thermocouple was positioned at the center of the chamber to monitor the indoor air temperature. Testing was conducted using two types of glass: ordinary glass with high solar transmittance (87.5%) and low-emissivity (Low-E) glass with high thermal reflectance (66.8%).

**Building energy-saving simulations.**

In EnergyPlus, a model house with dimensions of 8 m × 8 m × 3 m was constructed, incorporating four 2 m × 4 m windows centrally positioned on each wall. Simulations were conducted for three window configurations: ordinary glass window, low-emissivity (Low-E) window, and PTFE/PVA windows. The optical properties of these window types are detailed in Table S1. Climate data from 13 representative cities across various latitudes were utilized to evaluate the performance of the windows under diverse environmental conditions. The indoor thermal regulation provided by the smart windows was simulated in the absence of an HVAC system. For energy-saving assessments, a single HVAC system with a constant setpoint temperature of 22 °C was introduced, and the heating and cooling energy consumption for each configuration was recorded.

**Statistical Analysis**

The formulas for solar reflectance (*R_solar_*) and long-wave infrared transmittance (*T_LWIR_*) in Figure 3, Figure S2, Figure S5, and Figure S6 are as follows:

$R_{solar}=\frac{\int_{0.3 \mu m}^{2.5 \mu m} R\left( \lambda\right)E_{solar}\left( \lambda\right)d\lambda}{I_{solar}}$ (1)

$T_{LWIR}=\frac{\int_{8 \mu m}^{13 \mu m} T\left( \lambda\right)E_{bla}\left( \lambda\right)d\lambda}{I_{bla}}$ (2)

where:

*R(λ)* is the measured spectral reflectance, and *T(λ)* is the measured spectral transmittance.

*E_solar_(λ)* represents the AM 1.5G solar spectral irradiance.

*E_bla_(λ)* denotes the blackbody spectral irradiance at 300 K.

*I_so_*_lar_ and *I_bla_* correspond to the total solar irradiance and total blackbody irradiance, respectively.


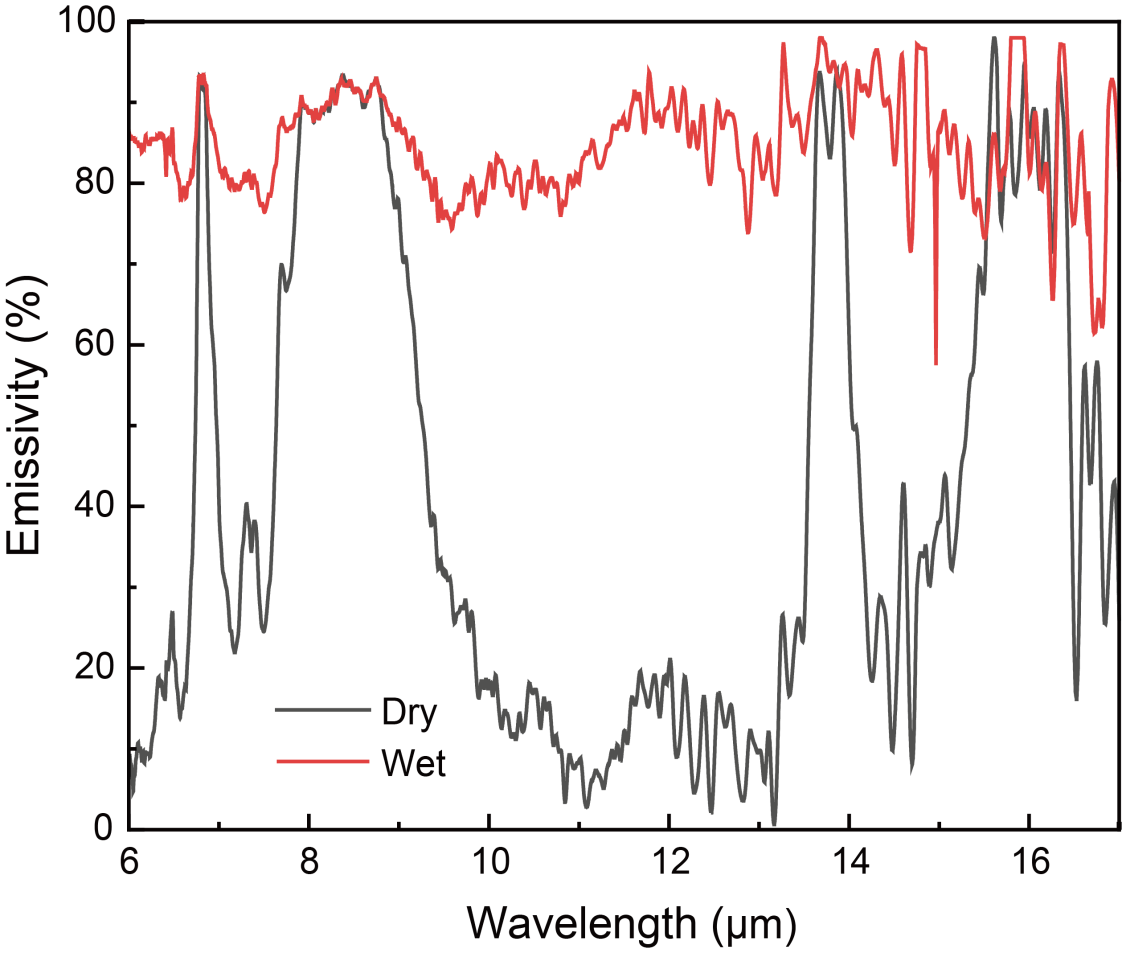


**Figure S1.** The IR emissivity (8-13 μm) of the composite membrane in wet and dry state.


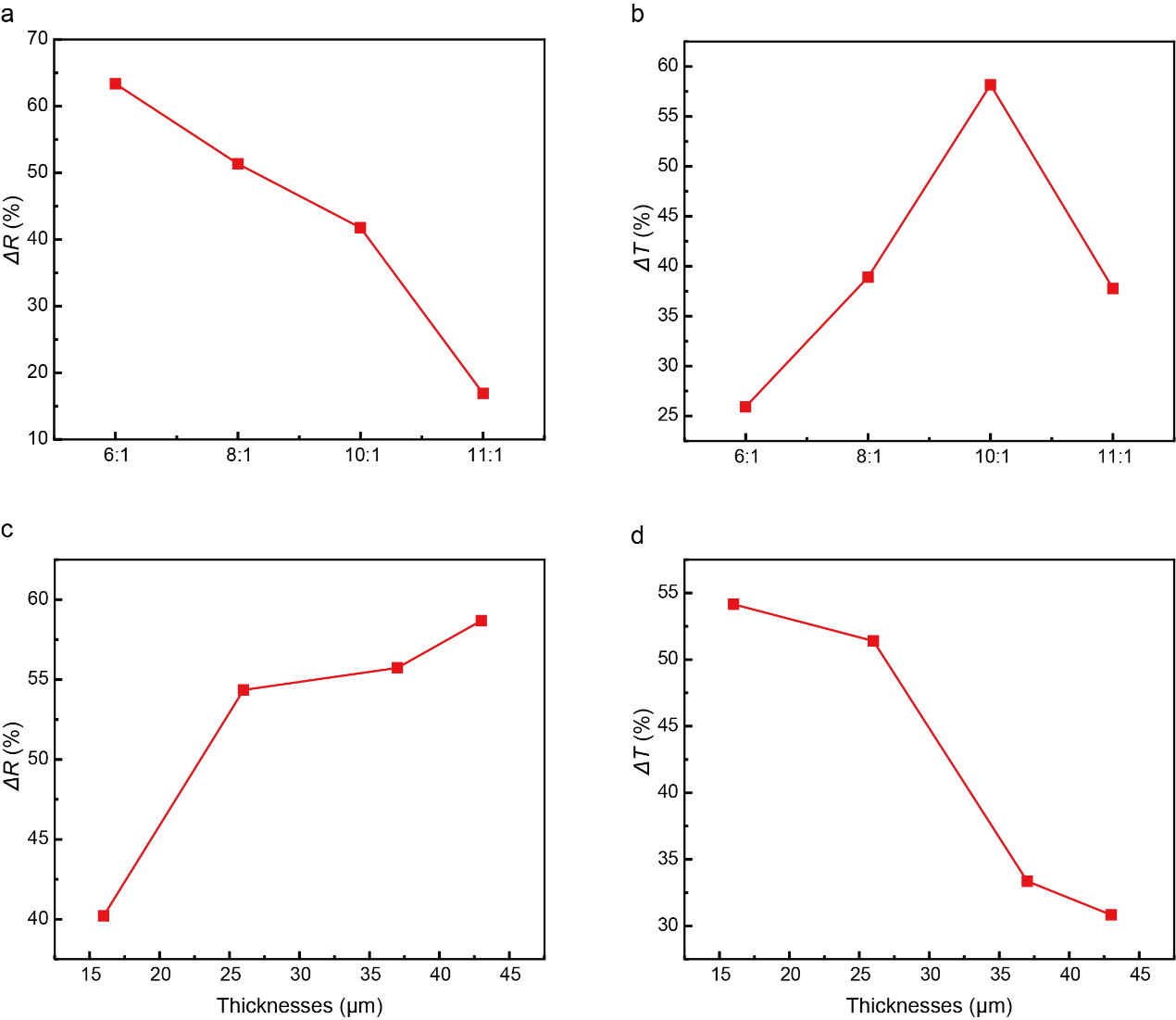


**Figure S2.** (a and b) Effect of the mass ratio of PTFE solution (60 wt%) and PVA solution (15 wt%) in the spinning solution on the solar band reflectance difference (a, Δ*R*) and long-wavelength infrared transmittance difference (b, Δ*T*) between dry and wet modes of composite membrane, respectively. (c and d) Effect of membrane thickness on the Δ*R* (c) and Δ*T* (d) between dry and wet modes of composite membrane, respectively.


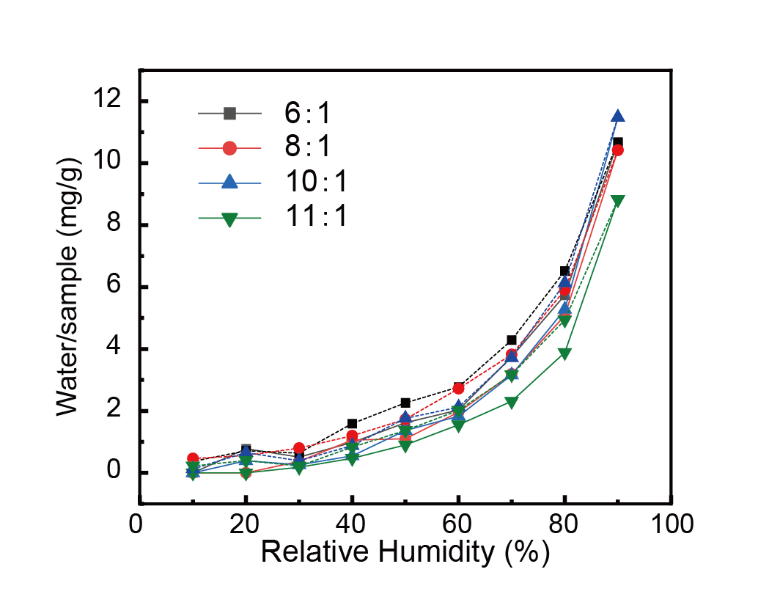


**Figure S3.** Dynamic water vapor adsorption and desorption curves of membranes with different mass ratios. The mass ratios between PTFE solution and PVA solution in the spinning solution were 6:1, 8:1, 10:1 and 11:1, respectively.


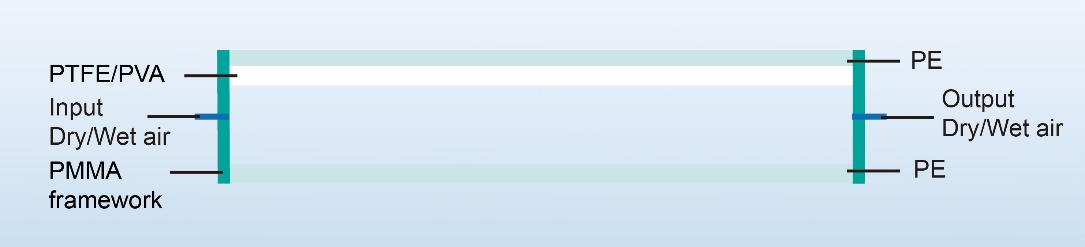


**Figure S4.** Schematic diagram of PTFE/PVA window. The PTFE/PVA membrane was directly fabricated onto a transparent polythene (PE) substrate and integrated with a transparent PE film using an acrylic frame. Dynamic transitions of membrane between the wet and dry modes were achieved by introducing humid or dry air through a pipeline connected to the frame. The smart window maintains complete hermetic sealing when no dry/wet air flow is applied.


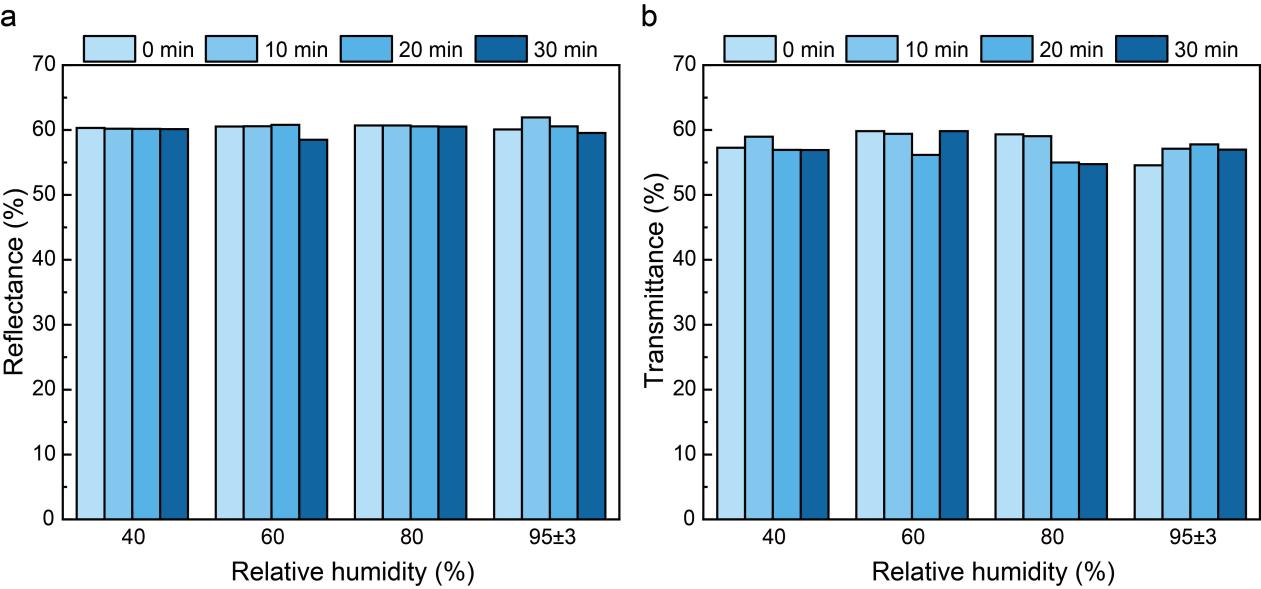


**Figure S5.** (a and b) Solar reflectance (a) and infrared transmittance (b) characteristics of PTFE/PVA composite membranes following 30-minute conditioning at different relative humidity levels.


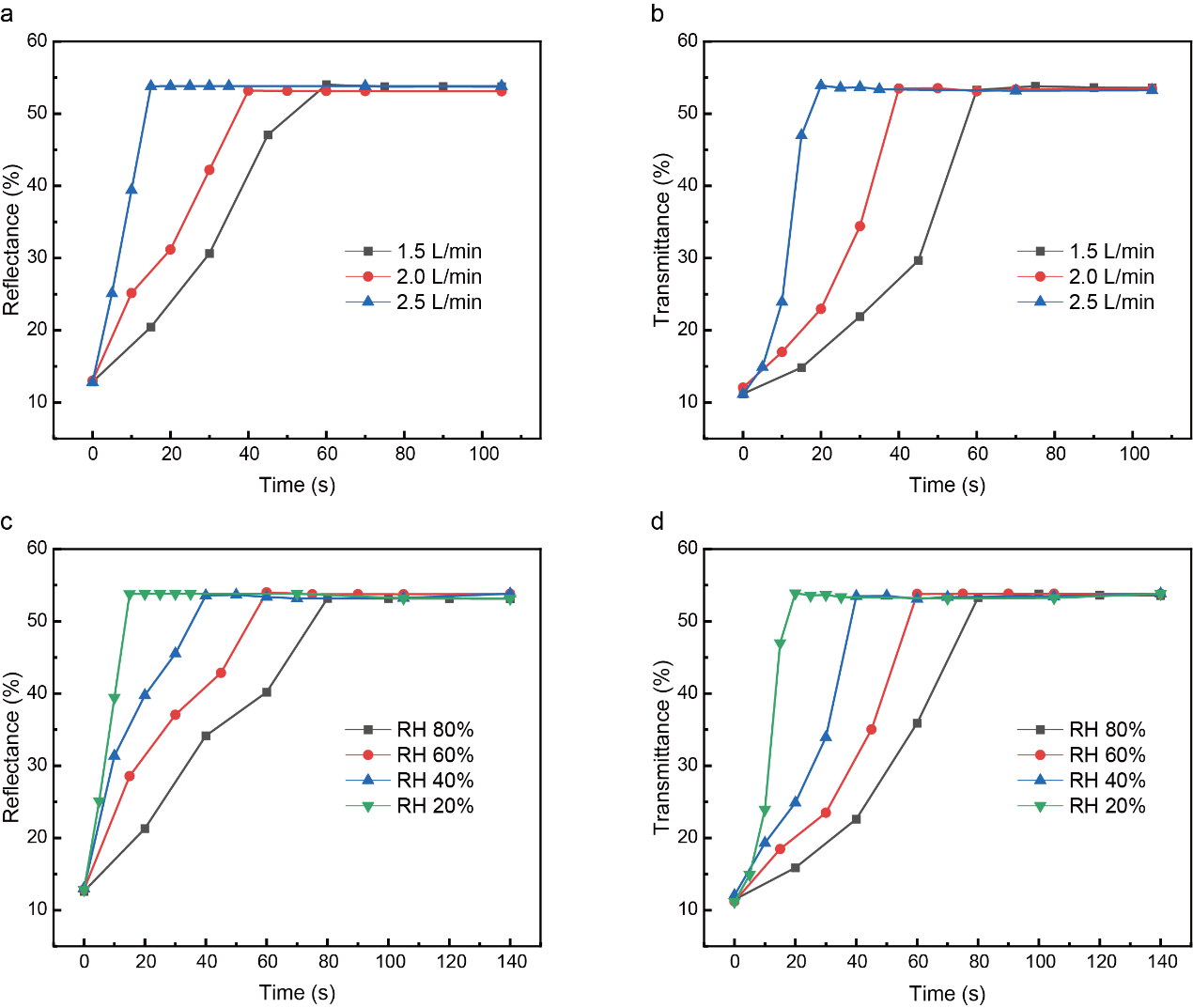


**Figure S6.** Continuous modulation performances of PTFE/PVA membrane. (a and b) Variation of the solar band reflectance (a) and the long-wavelength infrared transmittance (b) of the membrane from wet mode to dry mode under different dry air velocities, respectively. (c and d) Variation of the solar band reflectance (c) and the long-wavelength infrared transmittance (d) of the membrane from wet mode to dry mode under different relative humidity air, respectively. The air velocity was 2.0 L/min.


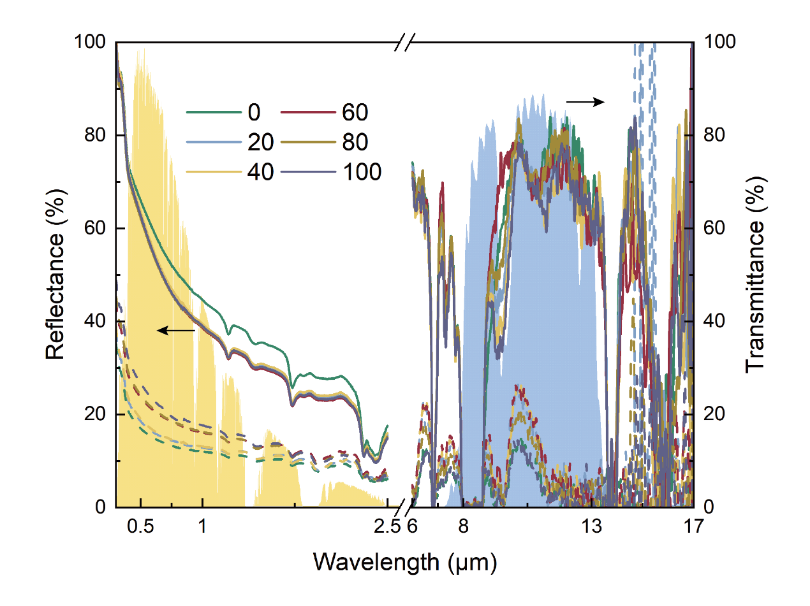


**Figure S7.** Spectra of PTFE/PVA windows during the drying-wetting cycles. The numbers (0, 20, 40, 60, 80 and 100) represent the number of cycles. The solid and dashed lines represent dry and wet modes of the PTFE/PVA window, respectively.


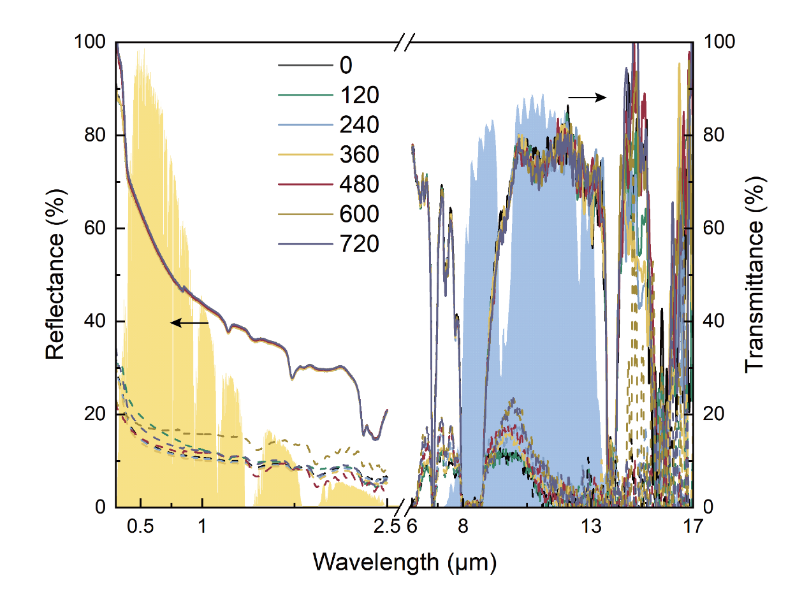


**Figure S8.** Spectra of the PTFE/PVA windows in long-term tests. The numbers (0, 120, 240, 360, 480, 600 and 720) represent the time in the test. The solid and dashed lines represent dry and wet modes of the PTFE/PVA window, respectively.


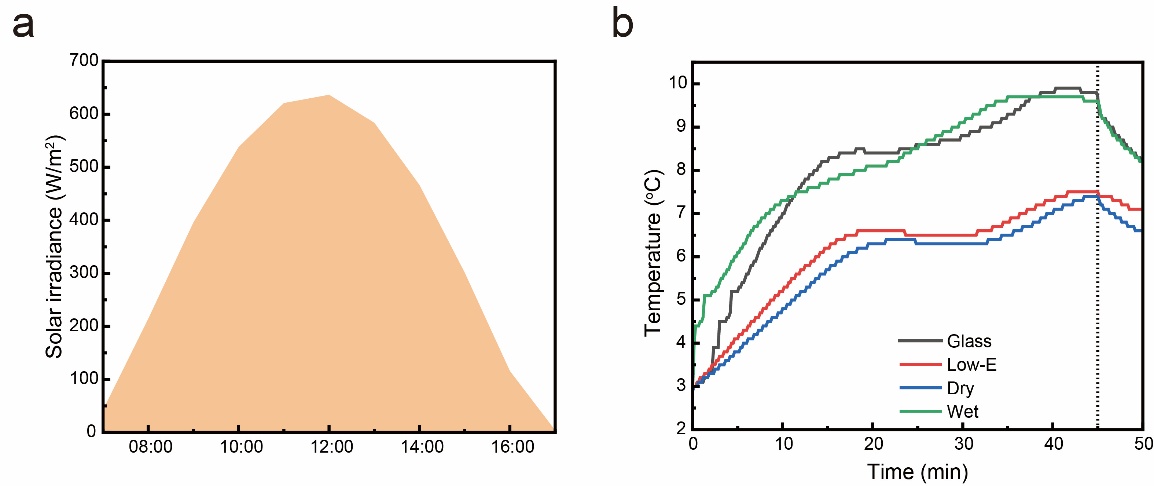


**Figure S9.** (a) Solar irradiance on January 15, 2025. (b) Indoor thermal management testing of chambers with different windows (low-E window, glass window and PTFE/PVA window with dry and wet modes) in simulated winter daytime. The simulated solar irradiance power was 400 W/m^2^.


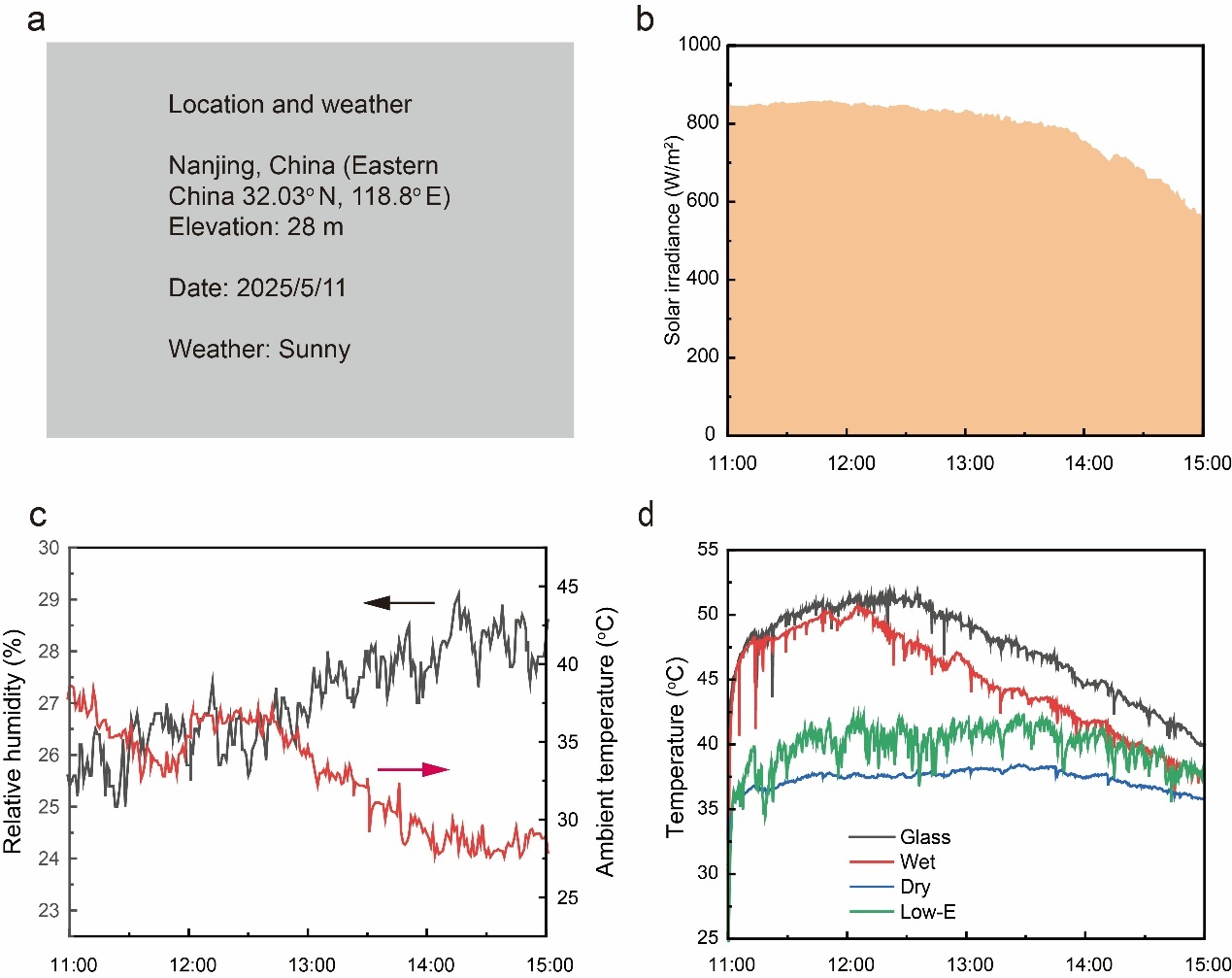


**Figure S10.** Outdoor temperature regulation tests of Humidity-responsive smart window (a) Topographic and weather information of the test locations. (b and c) Solar irradiance (b) and ambient temperature and relative humidity (c) on May 11, 2025. (d) Air temperature records of the chambers with different windows (Glass window, Low-E window, dry and wet modes of PTFE/PVA window) on May 11, 2025.


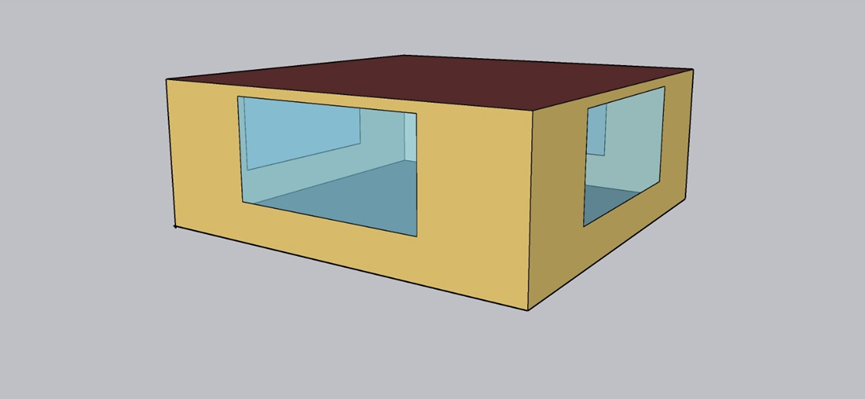


Figure S11. House model for energy saving simulation. House model has an area of 8 × 8 m^2^ and a height of 3 m. Four windows are incorporated into the structure, each measuring 4 × 2 m^2^.

**Table S1.** Thermal and optical data of different windows for energy-saving simulations. Data for PTFE/PVA window (Dry/Wet) is calculated based on the measured spectra. Data for glass and low-E are typical for commercial materials.

| Windows | Glass | Low-E | PTFE/PVA window  （Dry/Wet） |
| --- | --- | --- | --- |
| Solar  transmittance (%) | 87.5 | 30 | 39.9/ 77.7 |
| Solar reflectance(%) | 8.3 | 54.8 | 54.8/14.6 |
| Infrared transmittance | 0.01 | 0.006 | 0.574/0.044 |
| Infrared reflectance | 0.092 | 0.668 | 0.104/0.062 |
| Emissivity (outer) | 0.898 | 0.898 | 0.322/ 0.894 |
| Emissivity (inner) | 0.898 | 0.326 | 0.322/0.894 |
| k (W/(m·k)) | 0.733 | 0.747 | 0.0273/ 0.0295 |

**Table S2.** Comparative analysis of smart windows in temperature regulation.

| Smart window | Temperature regulation |
| --- | --- |
| PTFE/PVA window (Outdoor testing) | 13 ℃ |
| Solar and thermal regulatory (STR) window^[1]^ (Indoor testing) | 3 ℃ |
| Liquid flow electrochromic smart window (LF-ESW)^[2]^ (Outdoor testing) | 7.1 ℃ |
| PE film^[3]^ (Outdoor testing) | 10 ℃ |
| PTFE porous polymer coatings^[4]^ (Outdoor testing) | 18 ℃ |

**References**

[1] C. Lin, J. Hur, C. Y. Chao, G. Liu, S. Yao, W. Li, B. Huang, *Science Advances* **2022**, 8, eabn7359.

[2] Y. Huang, S. Wu, S. Zhao, Z. Guo, Z. Zhao, X. Wu, B. Wang, F. Wang, A. Xi, F. Lan, Y. Li, J. Xu, R. Li, Y. Zhao, R. Zhang, *Energy & Environmental Science* **2025**, 18, 1824.

[3] N. Guo, L. Yu, C. Shi, H. Yan, M. Chen, *Nano Letters* **2024**, 24, 1447.

[4] J. Mandal, M. Jia, A. Overvig, Y. Fu, E. Che, N. Yu, Y. Yang, *Joule* **2019**, 3, 3088.
